# Placenta Prævia

**Published:** 1889-11

**Authors:** P. K. Wortham

**Affiliations:** Meridian, Texas


For Daniel's Texas Medical Journal.

### PLACENTA PRÆVIA.

BY P. K. WORTHAM, M. D., MERIDIAN, TEXAS.

AN accident of so serious a nature as placenta prævia, or the implantation, in whole or in part, of the placenta over the mouth of the womb, although occuring but seldom, must have attracted the attention of accoucheurs from the earliest periods, when midwifery began to be practiced as an art, and its phenomena investigated with the least approach to scientific interest.

Accordingly we find tis condition noticed by writers of the seventeenth century as Guillemeau, Maurseeau, Astrue, Pugh and others; but these writers supposed that, when the placenta was found in this position, it had been separated from its normal site, and had fallen to the lower segment of the uterus.

Portal, who wrote in 1743, clearly describes this condition in minor cases. He was aware and taught that the placenta was originally attached to this site, and had not fallen there.

Leveret and Smellie, in 1752 gave minute descriptions of cases, with their pathology. But the great English accoucheur, Rigby, first in 1775, gave a full and clear view of this condition with distinct rules of practice.

He it was who gave the term, "unavoidable hemorrhage," to placenta prævia, a term that has been used by most writers as synonymous with placenta prævia, and enforced rules of practice even to our day.

The cause of this anomalous impantation of the placenta, though the source of much controversy and prolific of theories, can scarcely be said to be obscure or doubtful, and is given in the well known fact that it almost always occurs in the multiparæ.

At the time of ovulation, which is the menstrual act, the mucous membrane, which is the uterus, stimulated by the changes going on in the ovaries, becomes succulent, swollen, corrugated, closing the uterine cavity.

After conception, this condition is greatlv extended and intensified by the stimulus of the impregnated ovum—so much so, that, upon its arrival at the uterine extremity of the fallopian tube, it finds it obstructed by the swollen condition of the uterine mucous membrane; its further advance is arrested at this point, and its implantation at or near the fundus secured in all ordinary pregnancies.

After frequent child-bearing, the uterus loses its symmetry, and perfect coordination, remains larger—often more inactive, its cavity less perfectly filled by the corrugated mucous membrane, and the impregnated ovum, upon its arrival at the cavity of the uterus, falls to the lower segment, where it becomes fixed, just as it would have done in its normal site, or had it dropped into the cavity of the abdomen, as in extra uterine pregnancy.

In very rare cases of primipara, this condition may exist for want of correspondence of uterine with ovarian activity, or from some anomalous size or shape of the cavity. The ovum falling to, and becoming fixed upon the lower segment of the uterus, necessarily develops placenta at this site, which, rapidly developing in every direction, spreads over the internal os and the entire lower segment, forming placenta centralis; or, the ovum may be caught in a corrugation or fold of uterine mucous membrane some distance above the os, in which case only its lower margin may reach to, or over the os, giving placenta lateralis, or partial placenta prævia.

The causes, then of placenta prævia are not obscure or doubtful, for which fact we are not indebted to the many theories upon the subject, but to our advanced knowledge of the changes in the uterine mucous membrane during, and associated with, and caused by ovulation.

Symptoms: Most generally the first indication of placenta prævia is a gush of blood, without warning—and this without regard to the position, or mental state, or the duties the patient may be engaged in. Frequently this discharge corresponds with what would have been the menstrual period, had the woman not been pregnant, and is immediately dependent upon the periodical hyperæmia, continued, perhaps, in most women during pregnancy.

The first discharge—if the woman is not advanced more than five or six months, previous to which hemorrhage is rare——may be, and often is moderate in the quantity, ceases spontaneously, almost by the time the woman's attention is called to it; or if she is erect and engaged in her household duties, it ceases upon her assuming the supine position, and is readily controlled by simple remedies. But this is a respite only, as the hemorrhage is almost certain to return with increased violence within a few days; at farthest, at the menstrual period. Most generally it is not until near the close of gestation, or at the commencement of labor, that the hemorrhage first makes its appearance—often at this time with a frightful discharge of blood, that may in a few moments place the patient in iminent peril, or even destroy her, without prompt and scientific treatment. As a rule the nearer the women is to her full time, the greater the hemorrhage, which is greatest at the commencement of labor. Called to a case of hemorrhage during the latter months of pregnancy, a vaginal examination is absolutely necessary; and no feeling of delicacy upon the part of patient or accoucheur, nor fear of increasing the discharge by removing or breaking up clots, would justifiy us in neglecting it. Upon a vaginal examination, the diagnosis is seldom difficult or doubtful. If the os be sufficiently dilated to admit the finger, we will feel the placenta, either centrally or its margin inserted over the os, instead of the smooth membranes, If the finger cannot be inserted, the soft, boggy feel of the lower segment of the uterus, with the absence of the presenting part of the child, will readily determine the nature of the case.

### SOURCE OF HEMORRHAGE.

In one of the latest, as also one of the best works on midwifery, (Playfair), it is stated that it is pretty generally admitted by authorities that the immediate source of the hemorrhage is the lacerated utero-placental vessels. The blood in the placenta, as is well known, belongs to the child, was manufactured by the child, never was, and never becomes, a part of the mothers' blood. We have hemorrhages from the placenta, but, as is well known, it is then the child that perishes; whereas, in the hemorrhages of placenta prævia it is the mother that bleeds to death. Barnes' theory of placenta prævia, which has been pretty generally adopted, explains satisfactorily both these classes of cases. He describes the uterine cavity as divisible into three zones or regions; when the placenta is situated in the upper or middle of these zones, no separation or hemorrhage need occur during labor. When, however, it is situated partially or entirely in the lower or cervical zone, the expansion of the cervix during labor must produce more or less separation, and consequent loss of blood. As soon as the previous portion of the placenta is sufficiently separated, provided contraction of the uterine tissue be present to seal up the mouths of the vessels, hemorrhage no longer takes place. The placenta may not be entirely detached, but no further hemorrhage occurs in consequence of the remaining portion being ingrafted on the uterus beyond the region of unsafe attachment.

Dr. Duncan estimates the limit of the spontaneous detaching area to be a circle of 4½ inches in diameter, and that after the cervix has expanded to that extent no further separation or hemorrhage takes place. Barnes has sometimes observed that the hemorrhage has completely stopped when the os uteri opened to the size of the rim of a wine-glass or even less.

It will be seen, then that in this, as in every other form of puerperal hemorrhage the tendency of uterine contraction is to check the hemorrhage and that, provided the pains are sufficiently energetic, nature may be capable of stopping the flooding without artificial aid. It is but rarely, however, that she can be trusted for the purpose; and we shall presently see that these theoretical views have an important practical bearing on the subject of treatment.

Prognosis to both the mother and child is certainly grave in all cases of placenta prævia. Read in his treatise on placenta prævia estimates the maternal mortality, from the statistics of a large number of cases, as one in four and one-half cases and Churchill as one in three.

The mortality will of course, greatly depend on the treatment adopted. Out of sixty-four cases recorded by Barnes the deaths were six, or one in ten and two-thirds; under any circumstances the risks to the mother are very great.

TREATMENT: It is a practice established by high and multiplied authority, and sanctioned by success, to deliver women by art in all cases of dangerous hemorrhage without confiding in the resources of the constitution. Subsequent ages have but confirmed the practice stated by Denman, and the improvements have been, not evade it, but in the manner of its performance as the only safety to the woman as it was then is now to empty the uterus.

In all cases, then of hemorrhage, when the cause is ascertained to be placenta prævia, whether in the sixth or ninth month, or at term, the uterus must be emptied, as no reliance can be placed in position on a hard mattress, cold, acidulated drinks, opium, sugar of lead, cold applied to the vulva, plugging the vagina, etc., further than they may be used as immediate expediments, looking to the speedy delivery of the woman, or promotive of this end; and the physician who would trust his patient to these, with the view of prolonging gestation, is but trifling in the presence of a fearful danger.

Oberman: A contribution to the treatment of placenta prævia. (Archiv, of Gyn., XXX. I.)

The statistics utilized are from Crede's Clinic at Leipzig, there occurring sixty-four cases of placenta prævia, from the beginning of 1883 to the end of 1887; the maternal mortality being 11 per cent. and 55 per cent. for the children.

In forty-nine instances, the treatment consisted in combined version and slow extraction, and excluding one maternal death, owing to the hopelessness of the case when first seen, the maternal mortality by this method was 21 per cent., which is even better than that recorded by Hofmier from the same method—27 per cent.

The maternal mortality in fifteen cases treated by other methods was 33⅓ per cent. These data are offered as additional testimony in favor of the treatment of placenta prævia by means of version after Braxton Hicks' method, followed by slow extraction.

Nardman: Statistics and treatment of placenta Prævia, (Arch of Gyn, XXXII. I.)

The aim of this paper is to solve the question as to whether the preferable method of treatment is by combined version and slow extraction. Forty-five cases occurring at the Dresden Clinic in a total of 5,779 labors, are utilized.

Twelve cases were treated by tampon or colpeurynter, with or without rupture of the membranes, delivery being allowed to take place spontaneously, with a maternal mortality of o per cent. and an infantile of 16.16 per cent. excluding the cases where the ftœus was dead when first seen. In twenty-three cases, version was performed followed by immediate extraction, the maternal mortality being 17.3 per cent. and the infantile 5.8 per cent., excluding cases where the fœtal heart was never heard.

In six cases, version and slow extraction was the method of treatment, one mother dying of sepsis, and all the children being delivered dead. Although these data decidedly speak in favor of the first method of treatment, it should be noted that, in all the cases so treated, there was marginal insertion of the placenta, an insertion which does not expose the mother to the same risk as the total insertion. By the second method the greatest number of children were saved. N. concludes that this method is preferable in hospital practice, whilst in private practice version and slow extraction should be the rule, notwithstanding the excessive infantile mortality.

In case of placenta prævia marginalis, the tampon will answer both from the stand point of the mother and of the child.

